# Diversity of bacteriocins in the microbiome of the Tucuruí Hydroelectric Power Plant water reservoir and three-dimensional structure prediction of a zoocin

**DOI:** 10.1590/1678-4685-GMB-2021-0204

**Published:** 2022-01-05

**Authors:** Sávio S. Costa, Leticia, A. B. Lago, Artur Silva, Diego A. das Graças, Jerônimo Lameira, Rafael A. Baraúna

**Affiliations:** 1 Parque de Ciência e Tecnologia Guamá, Laboratório de Engenharia Biológica, Belém, PA, Brazil.; 2 Universidade Federal do Pará, Instituto de Ciências Exatas e Naturais, Laboratório de Planejamento e Desenvolvimento de Fármacos, Belém, PA, Brazil.

**Keywords:** Bacteriocin, whole metagenome sequencing, Zoocin, Tucuruí-HPP

## Abstract

Bacteriocins are antimicrobial peptides expressed by bacteria through ribosomal activity. In this study, we analyzed the diversity of bacteriocin-like genes in the Tucuruí-HPP using a whole-metagenome shotgun sequencing approach. Three layers of the water column were analyzed (photic, aphotic and sediment). Detection of bacteriocin-like genes was performed with blastx using the BAGEL4 database as subject sequences. In order to calculate the abundance of bacteriocin-like genes we also determined the number of 16S rRNA genes using blastn. Taxonomic analysis was performed using RAST server and the metagenome was assembled using IDBA-UD in order to recover the full sequence of a zoocin which had its three-dimensional structure determined. The photic zone presented the highest number of reads affiliated to bacteriocins. The most abundant bacteriocins were sonorensin, Klebicin D , pyocin and colicin. The zoocin model was composed of eight anti-parallel β-sheets and two α-helices with a Zn^2+^ ion in the active site. This model was considerably stable during 10 ns of molecular dynamics simulation. We observed a high diversity of bacteriocins in the Tucuruí-HPP, demonstrating that the environment is an inexhaustible source for prospecting these molecules. Finally, the zoocin model can be used for further studies of substrate binding and molecular mechanisms involving peptidoglycan degradation.

## Introduction

In the environment, several species of free-living microorganisms coexist and their adaptive success depends, in part, on the molecular mechanisms of defense and competition (Nes *et al.,* 1996; Quereda *et al.,* 2016). Among these mechanisms there are the so-called antimicrobial peptides (AMPs). They are synthesized in ribosomes and the gene clusters that encode AMPs are widely distributed in nature (Nissen-Meyer and Nes, 1997). AMP expression has already been reported in mammals, plants, insects, and bacteria (Hancock and Chapple, 1999). The AMPs produced by bacteria are narrow-spectrum anti-bacterial agents called bacteriocins. These peptides have activity against bacteria that are taxonomically related to the producing species. However, some broad-spectrum AMPs have already been described (Cleveland *et al.,* 2001). Some studies suggest that approximately 99% of bacterial species produce bacteriocins (Riley and Wertz, 2002).

The first bacteriocins characterized were produced by the model species *Escherichia coli* and were called colicins (Rehm and Lazdunski, 1988). Colicin acts by forming a voltage-dependent channel into the inner membrane of bacteria causing an imbalance of electrochemical gradient and, consequently, cell death. Colicin also acts as an endonuclease on DNA, rRNA, or tRNA of the target cells (Riley and Wertz, 2002). Several other bacteriocins have been described and studied since then as enterocin K1 (Ovchinnikov *et al.,* 2017), listeriolysin S (Quereda *et al.,* 2016), nisin O (Hatziioanou *et al.,* 2017), among others.

Different methods for the classification of bacteriocins were proposed (Klaenhammer 1993; Franz *et al.,* 2007; Zouhir *et al.,* 2010). Klaenhammer (1993) proposed the classification of bacteriocins produced by lactic acid bacteria (LAB) into four classes according to molecular weight, mechanism of action, and biochemical characteristics. This classification is one of the most used today. 

Bacteriocins have a wide range of application. The most successful applications are related to the food industry and agriculture (Snyder and Worobo, 2014). AMPs are a promising alternative to the use of chemical preservatives in food production (Chopra *et al.,* 2015). Nisin, a bacteriocin of class Ia according to the Klaenhammer classification, was one of the first AMPs to be commercialized as a natural preservative for foods under the name Nisaplin^™^. This product is currently commercialized in several countries around the world. The use of bacteriocins as an alternative to antibiotics is also widely discussed today (Cotter *et al.,* 2013). For example lacticin 3147 acts in synergy with polymyxin to inhibit Gram-negative bacteria such as *Cronobacter* and *E. coli* (Draper *et al.*, 2013). Additionally, several studies have analyzed the ability of bacteriocins to inhibit the formation of biofilms in order to assist in the clinical treatment of pathogenic biofilm-forming bacteria such as methicillin-resistant *Staphylococcus aureus*, *Staphylococcus epidermidis*, *Pseudomonas aeruginosa*, *Gardnerella vaginalis*, and *Streptococcus mutans* (Mathur *et al.,* 2018). Another promising medical application of bacteriocins is their cytotoxic activity against cancer cell lines (Baindara *et al.,* 2017).

Despite the well-established knowledge and the biotechnological application of bacteriocins, little is known about the diversity and distribution of these AMPs in environmental microbial communities. Most studies using a metagenomic approach to evaluate the diversity of bacteriocins are focused on host-associated microbiomes (Walsh *et al.,* 2015; Zheng *et al.,* 2015). In our study, we analyzed the diversity and abundance of bacteriocin-like genes in the microbial community of the Tucuruí Hydroelectric Power Plant (Tucuruí HPP) water reservoir, located in the state of Pará, Brazil, using a whole metagenome sequencing approach. Additionally, the metagenome was assembled and the three-dimensional structure of zoocin was determined using homology modeling and molecular dynamics. 

## Material and Methods

### Sampling and DNA extraction

Sampling was performed in the Tucuruí-HPP water reservoir in 2015. Tucuruí-HPP is located at the eastern Brazilian Amazonia, in the Tocantins river. Three layers of the water column were sampled: photic zone (water surface), aphotic zone (15 m deep), and sediment (3° 49′ 56″ S, 49° 38′ 59″ O). Twenty liters of water at each layer was sampled using a Van Dorn bottle and 3 g of sediment was collected using a Van Veen grab. Water samples were maintained in previously sterilized bottles and vacuum filtered on the same day of sampling. Nitrocellulose membranes of 0.22 µm pore size (Millipore, Danvers, USA) were used to retain microbial cells during filtration. These membranes were frozen in STE buffer (NaCl 5 M, Tris 1 M, EDTA 0,1 M) until DNA extraction. The sediment sample was added directly to a polypropylene tube containing STE buffer. DNA extraction was performed using the UltraClean^™^ Soil DNA kit (MoBio, Maryland, USA) according to the manufacturer’s protocol. DNA quantification was performed using a nano spectrophotometer and the quality of the extracted material was analyzed by electrophoresis in 1.5% agarose gels.

### Sequencing and data processing

Sequencing was performed in the Ion^™^ Proton platform (Thermo Fischer Scientific, Missouri, USA) with Ion PI chip v3 that generates reads with up to 200 bp and up to 10 Gb of genetic information per run. Reads were converted to the fastq format and filtered by quality (bases with Q<20 were discarded) and by size (reads less than 50 bp after quality filter were discarded). Data processing was performed using the FASTX-Toolkit (http://hannonlab.cshl.edu/fastx_toolkit). 

### Detection and abundance of bacteriocin-like genes

The method developed by Zheng et al. (2015) for detecting resistance genes in whole metagenome sequencing data was adapted to detect bacteriocin-like genes. First, all bacteriocin sequences present in the BAGEL4 database was downloaded (de Jong *et al.,* 2006; van Heel *et al.,* 2018). Redundant sequences were removed by searching for identical gene names within the dataset using an in-house awk command. Subsequently, a blastx of the metagenome reads against the sequences of the BAGEL4 database was performed using a script from BLAST+ (Altschul *et al.,* 1990) with the parameter max_target_ses = 1. Best hits were those with e-value < 0.001 and the highest coverage. 

A similar process was performed to find 16S rRNA gene sequences in the metagenome data but using blastn instead of blastx. The 16S rRNA subject sequences were downloaded from SILVA database (Quast *et al.,* 2013). The abundance of bacteriocin-like genes in the metagenome was calculated according to the adapted formula below (Li B *et al.,* 2015):

$$Abundance=\;\sum_{1}^{n}\frac{N_{bacteriocin\;sequences}\;X\;\frac{T_{read}}{T_{bacteriocin}}}{N_{16S\;rRNA\;sequences}\;X\;\frac{T_{read}}{T_{16S\;rRNA}}}$$

Where *N* represents the number of reads identified as bacteriocin sequences (*N*_*bacteriocin sequences*_) or 16S rRNA sequences (*N*_*16S rRNA sequences*_). *T*_*read*_ represents the average size (bp) of the metagenome reads identified as bacteriocins or 16S rRNA. Finally, *T*_*bacteriocin*_ and *T*_*16S rRNA*_ represents the average size of the genes that encode bacteriocins and 16S rRNA, respectively.

### Assembly and identification of complete genes

The metagenome assembly was performed with metaSPADES (Nurk *et al.,* 2017) Megathit (Li D *et al.,* 2015) and IDBA-UD (Peng *et al.,* 2012). The assembled metagenomes were compared using metaQUAST v2.2 (Mikheenko *et al.,* 2016). The assembly with the highest N50 was used to predict open reading frames (ORFs) using the software MetaGeneMark (Zhu *et al.,* 2010). Predicted genes were aligned against bacteriocin nucleotide sequences that have a three-dimensional structure available in the Protein Data Bank (PDB). The alignment was performed using blastn. Best hits were those with e-value < 0.001 and the highest coverage and identity.

### Taxonomic analysis and statistical graphs

Microbial diversity was analyzed in MG-RAST server (Meyer *et al.,* 2008). The output file was filtered to obtain only the reads affiliated to Bacteria domain. The pie chart (taxonomy analysis) and the ternary plot (bacteriocin distribution over the samples) were made in R (Team RC, 2013).

### Protein structure modeling

Comparative modeling is a well-known tool that is used to predict the three-dimensional structure of a given primary structure (target) based on its alignment to one or more proteins of known structure (templates) (Martí-Renom *et al.,* 2000). In this work, the Swiss-Model Workspace (Waterhouse *et al.*, 2018) was used to predict the zoocin structure. Initially, the zoocin primary structure was aligned against the sequence of zoocin from *Streptococcus equi* subspecies *zooepidemicus* 4881 with known structure (Protein Data Bank, PDB code: 5KVP) (Xing *et al.,* 2017) that was used as template. The model validation and analysis was performed through the Ramachandran plot (stereochemistry quality) and QMEAN (Benkert *et al.,* 2011) both available in Swiss-Model Server (Waterhouse *et al.,* 2018). VMD software (Humphrey *et al.,* 1996) was used to display the protein structures.

### Molecular dynamics simulations

The initial coordinates for the zoocin model were taken from the best prediction provided by the Swiss-Model server as the starting point for MD simulations. The system was solvated in a truncated octahedron TIP3P (Jorgensen *et al.,* 1983) water box. Five counter ions (in this case, Na^+^) were added to maintain electro-neutrality of each ligand-protein complex system. It is also important to point out that the standard protonation state at pH=7 was assigned to all ionizable residues, where the protonation states of all the residues of protein were carefully defined according to the PROPKA (Jorgensen *et al.,* 1983). All residues were considered in their standard forms at pH=7 in both systems. The AMBER 18 suite of programs (Case *et al.*, 2018) force field was used to perform MD simulations, where the SHAKE algorithm (Andersen, 1983) was used to maintain all the bonds at their equilibrium distances. Initially, the hydrogens, water molecules, and ions were minimized using 10,000 cycles of steepest descent and conjugate gradient algorithms (Hestenes and Stiefel, 1952). Then, the whole system was heated through several heating steps. Finally, we performed 10 ns of molecular dynamics *(*MD) simulation using the NPT ensemble. The parameter for the Zn center was built using MCPB.py, which has been developed to build force fields for the simulation of metal complexes employing the bonded model approach (Li and Merz, 2016).

### Data availability

These sequence data have been submitted to the EBI database under the accession numbers ERS1560860, ERS1560861 and ERS1562591.

## Results and Discussion

### Diversity of bacteriocins

**Figure 1 -:**
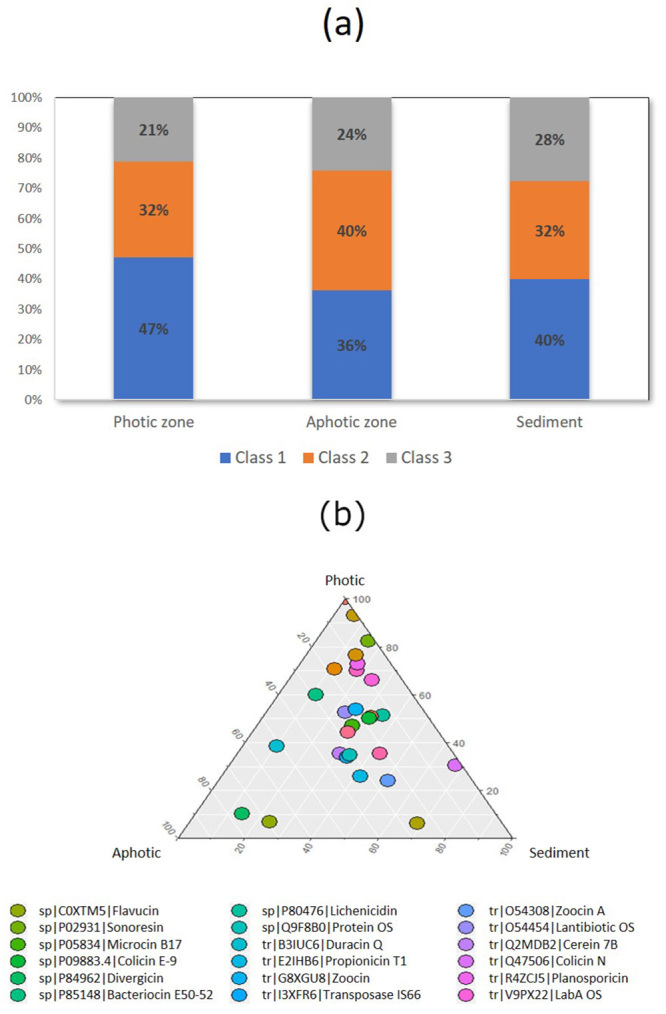
Bacteriocins in the Tucurui HPP water reservoir. (a) Percentage of class 1, 2 and 3 bacteriocins in the three layers analyzed. The total number of reads identified as bacteriocins was used to calculate the percentage of each class. (b) Ternary plot showing the distribution of eighteen bacteriocins in the three zones analyzed. Bacteriocins were widely distributed along the water column with a slightly higher prevalence in the photic zone.

Table S1 summarizes the sequencing data. The average size of the reads was 158 bp. A total of 18,879,156, 12,964,808, and 29,651,925 reads were obtained in the photic, aphotic and sediment zones, respectively. The percentage of reads identified as bacteriocins in each layer of the water column is presented in Figure 1. BAGEL4 database had a total of 491, 231, and 97 bacteriocin sequences from class 1, 2, and 3, respectively (http://bagel4.molgenrug.nl/). All these bacteriocins available in BAGEL4 had at least one affiliated read, which demonstrates a high diversity of AMPs in the Tucuruí-HPP. Class 1 and 2 were the most prevalent along the water column (Figure 1a). Ternary plot shows that the bacteriocin genes are widely distributed along the water column, with a slight predominance of some genes in the photic zone (Figure 1b). The photic zone showed the highest number of reads identified as bacteriocins (Table 1). Sonorensin, Klebicin D, Zoocin A, Pyocin and Colicin were the most abundant bacteriocins in the water column (Table 1).

**Table 1 -:** The five most abundant bacteriocins in the photic, aphotic and sediment zones of the Tucuruí-HPP water reservoir. Abundancy is calculated according to the formula provided in Methods, adapted from Li B *et al.* (2015).

Photic zone				
Bacteriocin	Abundancy	Class	# of affiliated reads	Bacterial species
Sonorensin	0.14	C1	14,582	*Bacillus sonorensis*
Klebicin D	0.006423	C3	3,192	*Klebsiella oxytoca*
Colicin	0.003271	C3	1,790	*Escherichia coli*
Zoocin A	0.00304	C3	1,574	*Sinorhizobium fredii*
Pyocin	0.002538	C3	1,490	*Pseudomonas aeruginosa*
Total	0.183741		39,080	
Aphotic zone				
Bacteriocin	Abundancy	Class	# of affiliated reads	Bacterial species
Sonorensin	0.027634	C1	2,386	*Bacillus sonorensis*
Zoocin A	0.001285	C3	1,559	*Streptococcus equi*
Transposase IS66	0.001285	C3	1,500	*Sinorhizobium fredii*
Klebicin D	0.001284	C3	1,064	*Klebsiella oxytoca*
Colicin E9	0.001284	C3	785	*Escherichia coli*
Total	0.039187		13,450	
Sediment				
Bacteriocin	Abundancy	Class	# of affiliated reads	Bacterial species
Sonorensin	0.037593	C1	3,823	*Bacillus sonorensis*
Zoocin A	0.001091	C3	3,112	*Streptococcus equi*
Klebicin D	0.001091	C3	2,039	*Klebsiella oxytoca*
Pyocin AP41	0.001091	C3	1,949	*Pseudomonas aeruginosa*
Colicin E7	0.001091	C3	1,537	*Escherichia coli*
Total	0.049592		25,456	

Class 1 bacteriocins have a small molecular weight and a range of applications in the food and veterinary industry (van Kraaij *et al.,* 1999; Cotter *et al.,* 2005). One of its main representatives are the lantibiotics. Class 2 bacteriocins are thermostable low-molecular weight peptides that in some cases are resistant to proteases and acidic stress (Netz *et al.,* 2002). The heat stability can be a major factor for the higher abundance of this class in microbial communities of tropical environments such as Amazonia. To the best of our knowledge, this is the first study to describe the diversity of bacteriocin-like genes in microbial communities of a tropical zone using metagenomics. Our data demonstrates the potential of free-living and uncultured bacteria as a source of AMPs with biotechnological application. The high diversity of bacteriocins in the Tucurui-HPP reservoir may be associated with several factors including the high availability of organic matter or inorganic compounds in the reservoir due to flooding caused by the construction of the dam. The process may have significantly altered the microbiome composition and led to the production of AMPs. This process was demonstrated in the Lancang River, China, were the bacterial diversity was significantly modified due to the construction of the dam (Shi *et al.,* 2013).

Sonorensin was the most abundant bacteriocin in the analyzed sites (Table 1). It is classified in the class 1 and is produced by *Bacillus sonorensis*. Gram-positive bacteria, specially from *Bacillus* genus, have attracted attention due to their AMPs. Sonorensin was effective in slowing down food spoilage by preventing *Staphylococcus aureus* biofilm formation (Chopra *et al.,* 2014 *,* 2015). The bacteriocin was added in a bioactive packaging film which was kept in contact with the food. Additionally, sonorensin was also effective against dormant cells of *Escherichia coli* and *S. aureus* (Chopra *et al.,* 2015).

Colicin was also found in high abundancy in the analyzed sites (Table 1). Colicin was discovered in 1925 and is one of the most studied bacteriocins with more than 30 types characterized so far (Cursino *et al.,* 2006; Dobson *et al.,* 2012) This bacteriocin binds to a specific cell surface receptor and is subsequently translocated across the susceptible cell’s membrane. Cell death is induced by depletion of the proton motive force. Other mechanisms of action are also observed such as DNase and RNase activity within susceptible cell (Montville *et al.,* 1995; Yang *et al.,* 2014; Atanaskovic and Kleanthous, 2019). About 20 types of colicin-producing *E. coli* were effective in inhibiting the growth of Shiga toxin-producing *E. coli* (STEC), a bacterium responsible for cases of diarrhea and hemolytic-uremic syndrome (Jordi *et al.,* 2001). Interestingly, in addition to the antimicrobial activity, colicin was capable of inhibiting growth of tumor cells (Yang *et al.,* 2014) an activity also observed in other bacteriocins such as Nisin ZP from *Lactococcus lactis* (Kamarajan *et al.,* 2015) and Pyocin S2 from *Pseudomonas aeruginosa* (Abdi-Ali *et al.,* 2004). Pyocins, like other bacteriocins, are seen as an alternative to antibiotics. Thus, this bacteriocin has been widely studied due to the increasing number of multi-drug resistant Gram-negative pathogens (McCaughey *et al.,* 2016).

Zoocin is a thermolabile high molecular weight bacteriocin that belongs to the class III, bacteriolisins subgroup (Mills et al., 2017), that acts by degrading the peptidoglycan of target cells (Khan *et al.*, 2013). Zoocin is commonly expressed by *Streptococcus equi* subsp. *zooepidemicus* (Mills *et al.*, 2017). Zoocin-producing bacteria contains two genes: *zooA*, that encodes the bacteriocin; and *zif*, that encodes a resistance gene (Beatson et al., 1998). Some of those genes are also associated to β-lactams resistance (Health et al., 2004). In our data, zoocin was detected in all layers of the water column. Due to its biotechnological importance and availability of a template in the Protein Data Bank (PDB), the *zooA* gene was recovered from the assembled metagenome and used to predict the three-dimensional structure of this bacteriocin.

**Figure 2 -:**
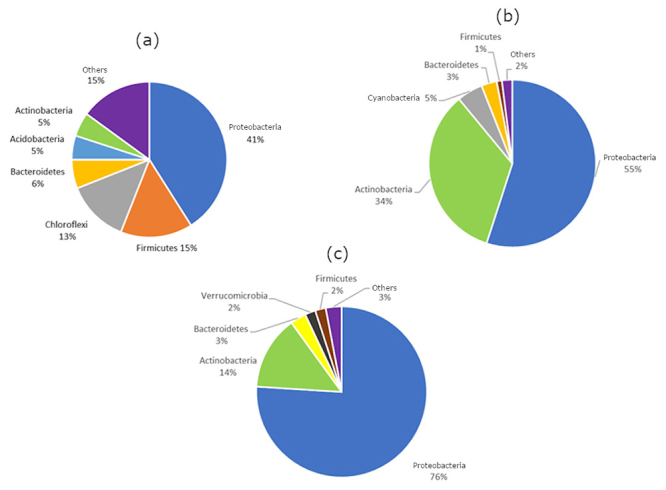
Diversity of bacterial phyla found in the three layers of the Tucuruí-HPP reservoir water column. Identification was performed by comparing the 16S rRNA reads with the MG-RAST server. (a) sediment; (b) aphotic zone; (c) photic zone.

We evaluated the bacterial diversity along the water column using MG-RAST server (Figure 2). We found several phyla of bacteriocin-producing species such as Proteobacteria, Actinobacteria and Firmicutes (Figure 2). Proteobacteria is a phylum composed of metabolic diverse Gram-negative species, mostly mesophilic, but with some thermophilic and psychrophilic species as well (Kersters *et al.,* 2006). It is one of the most common phyla of bacteria found in aquatic environments (Geraldes *et al.,* 2012). One of the most abundant bacteriocins along the water column, Klebicin D, is produced by *Klebsiella* spp., a species classified in the Proteobacteria phylum (Table 1). Similarly, sonorensin is produced by *Bacillus sonorensis* (Table 1), a species classified in the Firmicutes phylum, one of the most abundant phyla in the sediment of the reservoir (Figure 2). Lactic acid bacteria belong to the phylum Firmicutes and are very important in prospecting for bacteriocins. They are the main producers of lantibiotics and include species from the genera *Streptococcus*, *Lactobacillus*, *Lactococcus* and *Aerococcus*. These bacteria are mostly anaerobic facultative which explains their predominance in the sediment (Figure 2). Chloroflexi was also abundant in the sediment and several reads were affiliated to Cyanobacteria in the aphotic zone, which indicates incidence of light in deeper regions of the reservoir (Figure 2).

As previously explained, based on the relative abundance, biotechnological potential, and availability of a template, the three-dimensional structure of zoocin was used for homology modeling and molecular dynamics. In order to obtain the complete sequence of the bacteriocin, three assembly software were tested: IDBA, SPAdes and MEGAHIT (Table S2). IDBA was the software selected due to the higher number of assembled contigs and a higher N50 (Table S2). 

### Zoocin model

**Figure 3 -:**
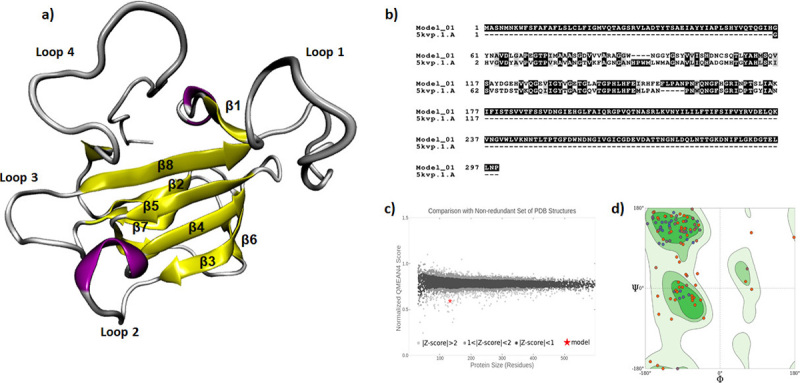
Comparative modeling results for Zoocin A. (a) Model of zoocin obtained from Swiss-Model Workspace (Waterhouse *et al.* 2018) (b) alignment sequence of Zoocin N-terminus and with the Zoocin A from *Streptococcus equi* subspecies *zooepidemicus* strain 4881 (c) QMEAN plot for the model (d) Stereochemical quality of the model from homology modeling with Swiss-Model Workspace (Waterhouse *et al.* 2018).

As commented in the methods section, the three-dimensional structure of zoocin was predicted by the Swiss-Model Workspace (Waterhouse *et al.,* 2018), using zoocin A from *Streptococcus equi* subspecies *zooepidemicus* strain 4881 as template (PDB code: 5KVP) (Xing *et al.,* 2017). It is worth noting that the model obtained corresponds to the catalytic domain of zoocin, which comprise residues from 60 to 176 (Figure 3a), where the target-template sequence identity is 47%. The stereochemical quality of the proposed model was evaluated using the PROCHECK tool. The location of the model in the QMEAN plot indicates the reliability of the structure prediction (Figure 3c). In, addition, Ramachandran plot showed that the model presents the most of residues in highly favorable regions (Figure 3d).

**Figure 4 -:**
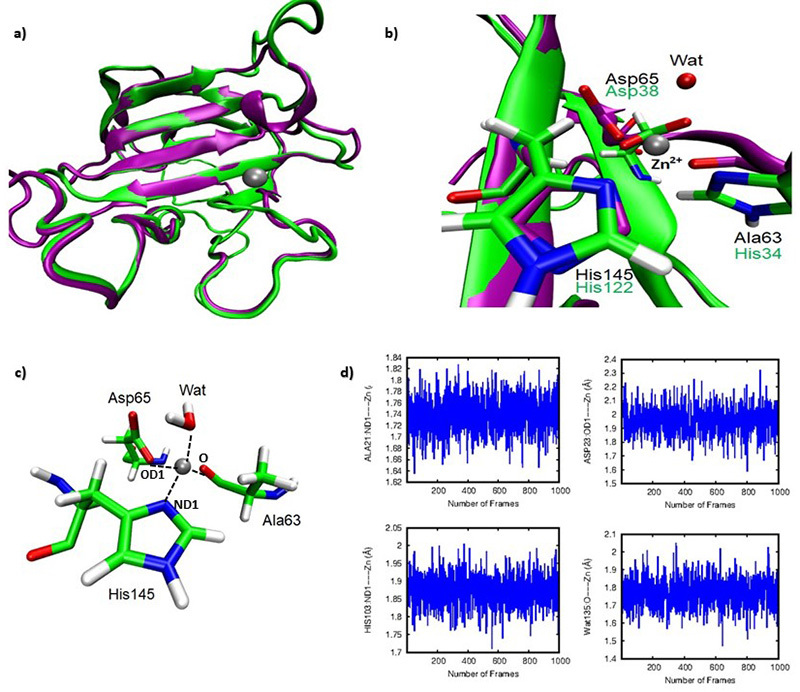
Molecular Dynamics results for Zoocin A. (a) Superposition of the zoocin model (purple) and zoocin A from *Streptococcus equi* subspecies zooepidemicus strain 4881 (green). (b) Position of residues His145, Asp65, Ala63 and a water molecule forming a tetrahedral coordination with Zn^2+^ in the active site. (c) Key distances for Zn^2+^ center computed during simulation. (d) Root-mean-square deviation (RMSD) plot computed during 10 ns of MD simulations shows a good stability for the Zoocin A model.

The Zoocin model obtained from comparative modeling is composed of eight anti-parallel β-sheets (β1- β8) and two α-helices (α1 and α2). It is worth to note that the template presents one α-helix. Additionally, there is a Zn^2+^ in the active site of the template (Figure 4a). Therefore, we placed a Zn^2+^ ion in the active site of our model, where it is possible to observe a formation of a metal center with a tetrahedral coordination with His145, Asp65, Ala63 and a water molecule (Figure 4b). Note that the position of His34 from the model match with Tyr61 in the target in the primary alignment. However, Tyr61 is 5.8 Å way from Zn^2+^ ion. In the tertiary structure, the oxygen atom of Ala63 backbone is in close proximity to Zn^2+^ ion, which suggest that Ala63 residue coordinates with this ion in active site of zoocin (Figure 4c). 

To explore the protein flexibility in solution and optimize the model of zoocin, we have performed 10 ns of MD simulations and analyzed the Root-mean-square deviation (RMSD) graph for the zoocin model structure taking as reference the Cα atoms of the initial model structure. The RMSD plot computed during 10 ns of MD simulations (Figure S1) shows that the model of zoocin has considerable stability (Figure 4d). The computed distances for Zn^2+^ center and the MD results demonstrate that His145, Asp65, Ala63 and a water are positioned to form a tetrahedral coordination with Zn^2+^ in the active site of zoocin. The computed average distance corresponded to 1.9, 1.9, 1.7 and 1.8 Å for tetrahedral coordination involving Zn^2+^ ion and His145, Asp65, Ala63 and a water molecule, respectively. These results are in agreement with experimental data for other endopeptidases of the lysostaphin family (Raulinaitis *et al.,* 2017), where observed distances correspond to 1.9 and 1.8 Å for ND1 of histidine and OD1 of aspartate, respectively in the active site of LytU from the lysostaphin family (Raulinaitis *et al.,* 2017). Overall, the model obtained for zoocin can be used to study the binding mode of the substrate and the molecular catalytic mechanism involving the degradation of peptidoglycan.

## Conclusions

Most of the terrestrial biomass is formed by microorganisms, which can be found in almost all environments. To help in the environmental adaptation, several microorganisms produce antimicrobial peptides, such as bacteriocins. Thus, the description of the diversity of these peptides in man-made environments such as the Tucuruí HPP brings us new insights about the ecology and application of these biological products. This work was able to describe the abundance of these peptides in three samples from Tucuruí-HPP water reservoir. A high diversity was observed, where all bacteriocins present in the BAGEL database were found in the three samples analyzed. The most abundant bacteriocins were Klebsin D, Zoocin A, Piocin and Sonoresin. A three-dimensional structure of Zoocin A was obtained. The model can now be used in several studies such as molecular docking for substrate binding analysis and molecular mechanism of peptidoglycan degradation. Bacterial taxonomic diversity was also evaluated. Most of the bacteriocins found such as Klebsin D and Sonoresin are produced by bacterial species classified in the most abundant phyla. This was one of the first studies of prospecting bacteriocin genes in the environment using a whole metagenomic sequencing approach.

## Supplementary material

The following online material is available for this article:

Table S1 - Amount, mean size and standard deviation of the sequencing raw data.

Table S2 - Assembly statistical data of the photic zone.

Figure S1 - RMSD plot computed along 10 ns of MD simulations for zoocin structure.
