# Supplementary material for: Diversity of bacteriocins in the microbiome of the Tucuruí Hydroelectric Power Plant water reservoir and three-dimensional structure prediction of a zoocin
Source: Genet Mol Biol. 2022 Jan 5;45(1):e20210204. doi: 10.1590/1678-4685-GMB-2021-0204 (PMC8762718; doi:10.1590/1678-4685-GMB-2021-0204)
Supplement: Table S1 - [file 1415-4757-GMB-45-1-e20210204-s1.pdf]

Genetics and Molecular Biology
https://doi.org/10.1590/1678-4685-GMB-2021-0204

Supplementary Material to "Diversity of bacteriocins in the microbiome of the Tucuruí Hydroelectric Power Plant water reservoir and three-dimensional structure prediction of a zoocin"

Table S1 - Amount, mean size and standard deviation of the sequencing raw data.

	Amount of reads	Mean size of reads	Standart deviation
Photic	18.879.156	163.29 bp	23.39 bp
Aphotic	12.964.808	152.27 bp	44.90 bp
Sediment	29.651.925	159.30 bp	53.77 bp
