# Supplementary material for: Diversity of bacteriocins in the microbiome of the Tucuruí Hydroelectric Power Plant water reservoir and three-dimensional structure prediction of a zoocin
Source: Genet Mol Biol. 2022 Jan 5;45(1):e20210204. doi: 10.1590/1678-4685-GMB-2021-0204 (PMC8762718; doi:10.1590/1678-4685-GMB-2021-0204)
Supplement: Table S2 - [file 1415-4757-GMB-45-1-e20210204-s2.pdf]

Genetics and Molecular Biology
https://doi.org/10.1590/1678-4685-GMB-2021-0204

Supplementary Material to "Diversity of bacteriocins in the microbiome of the Tucuruí Hydroelectric Power Plant water reservoir and three-dimensional structure prediction of a zoocin"

Table S2 - Assembly statistical data of the photic zone. IDBA was selected for the metagenome assembly due to the higher number of assembled contigs and a higher N50. Complete sequences of sonorensin and zoocin were found after assembly with IDBA.

Statistics	IDBA	Spades	MEGAHIT
contigs (>= 100bp)	131.388	834.857	1.526
contigs (>= 1000bp)	10	0	2
Largest contig	1754	531	1.520
Total length	135.540	531	7.221
Total length (>= 100)	12.351.231	33.208.642	373.515
Total length (>= 1000)	12.628	0	2.961
N50	590	531	755
N75	545	531	558
