# Supplementary material for: Diversity of bacteriocins in the microbiome of the Tucuruí Hydroelectric Power Plant water reservoir and three-dimensional structure prediction of a zoocin
Source: Genet Mol Biol. 2022 Jan 5;45(1):e20210204. doi: 10.1590/1678-4685-GMB-2021-0204 (PMC8762718; doi:10.1590/1678-4685-GMB-2021-0204)

Genetics and Molecular Biology
https://doi.org/10.1590/1678-4685-GMB-2021-0204

Supplementary Material to "Diversity of bacteriocins in the microbiome of the Tucuruí Hydroelectric Power Plant water reservoir and three-dimensional structure prediction of a zoocin"

Figure S1 - RMSD plot computed along 10 ns of MD simulations for zoocin structure.

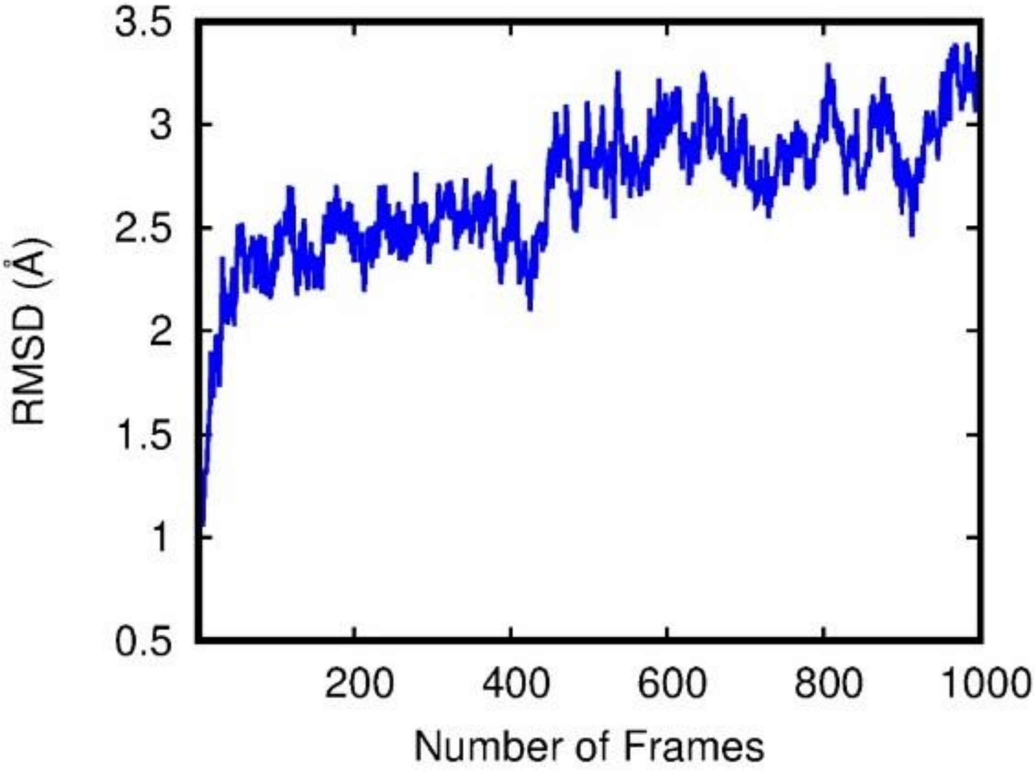

Supplement: Figure S1 - [file 1415-4757-GMB-45-1-e20210204-s3.pdf]
